# Multiple thread ligations versus stapled hemorrhoidopexy on operative outcomes of grade III hemorrhoids: A retrospective cohort study

**DOI:** 10.3389/fmed.2023.1156328

**Published:** 2023-03-28

**Authors:** Xile Zhou, Fanlong Liu, Caizhao Lin, Wenbin Chen, Jiahe Xu

**Affiliations:** Department of Colorectal Surgery, School of Medicine, The First Affiliated Hospital, Zhejiang University, Hangzhou, Zhejiang, China

**Keywords:** hemorrhoids, ligation, stapled hemorrhoidopexy, cohort study, propensity score matching

## Abstract

**Object:**

Controversy remains regarding the safety and efficacy of hemorrhoid ligation and stapled hemorrhoidopexy (SH) in the treatment of hemorrhoids. The study was to explore the operative outcomes of patients underwent multiple thread ligations (MTL) with SH for the management of grade III hemorrhoids.

**Methods:**

This cohort study included patients who underwent MTL (MTL group, 128 cases) or SH (SH group, 141 cases) for grade III hemorrhoids between June 2019 and May 2021. A total of 115 patients in MTL group and 115 patients in SH group were finally included by propensity score matching with a ratio of 1:1. The primary outcome was the recurrence of prolapse within 6 months. Secondary outcomes were operative time, post-operative pain scores, hospital stay, the incidence of complications, Wexner incontinence score, and quality of life of patients with constipation at 6 months post procedure.

**Results:**

Multiple thread ligations and SH resulted in comparable recurrence within 6 months of follow-up, with five and seven cases of recurrence, respectively, (*P* = 0.352). The two groups had comparable outcomes in terms of post-operative pain, hospital stay, Wexner incontinence scores, and constipation-related quality of life (all *P* > 0.05). The median operative time was 16 min (15–18 min) in the MTL group versus 25 min (16–33 min) in the SH group (*P* < 0.01). Univariate analysis showed that the MTL technique had a lower risk of postoperative bleeding than that with the SH technique (*P* < 0.05).

**Conclusion:**

The study indicated that the MTL technique might achieve comparable operative outcomes compared with the SH technique for the management of grade III hemorrhoids, nevertheless, MTL seemed to be associated with less risk of surgical bleeding than SH.

## Introduction

Hemorrhoidal disease occurs due to the enlargement and/or sliding of hemorrhoidal tissue resulting in symptoms and complications (1). The symptoms may include bleeding, pain, pruritus, fecal seepage, prolapse, and mucus discharge (2). Data on the prevalence of symptomatic hemorrhoids is scare (3), but they are thought to affect a considerable proportion of adults (4). Treatment depends on their severity (4). Conservative treatments such as dietary and lifestyle modifications to achieve a regular defecation with soft stool or oral phlebotonics to control symptoms are effective in the majority of patients in the early stages of the disease (5). While topical medications can also be beneficial to many patients (5). When intervention is required, there are many therapeutic options, including cryotherapy, infrared coagulation, sclerotherapy, rubber band ligation (RBL), doppler-guided hemorrhoidal dearterialization, hemorrhoidectomy, and various procedures of mucopexy and hemorrhoidopexy (6).

Hemorrhoidectomy has been strongly recommended for patients with grade III hemorrhoids (7, 8). However, conventional excisional hemorrhoidectomy, either open or closed, has disadvantages of high pain scores, high complication rates, and perceived recurrence (9). Therefore, although excisional hemorrhoidectomy remains an important option for advanced hemorrhoids and complicated hemorrhoids, minimally invasive operations are preferred when appropriate (9).

Stapled hemorrhoidopexy (SH) is associated with low pain scores and fast recovery compared with conventional hemorrhoidectomy (10). Nevertheless, SH may cause some unacceptable complications, such as persistent proctalgia due to retained staples, (11) new-onset fecal urgency, difficulty in defecation, or postoperative anorectal stenosis (12), even some cases of death have been reported previously (13). RBL has been the preferred therapeutic option for grade I/II hemorrhoids after the failure of basic treatment and is safe and simple (7, 8). However, for grade III hemorrhoids, the routine use of RBL in preference to SH remains controversial and a previous study has shown that SH has significantly better outcomes than RBL (14). As rubber bands are prone to fall off in the early stage in cases of multiple banding, elastic thread ligation may become a promising alternative.

This study aimed to compare the operative outcomes of patients underwent multiple thread ligations (MTL) with the SH technique for the management of grade III hemorrhoids.

## Patients and methods

### Study design and patients

This cohort study included patients with grade III hemorrhoids treated in the department of colorectal surgery in the first affiliated hospital of Zhejiang University between June 2019 and May 2021. The inclusion criteria were patients: (1) with a diagnosis of grade III hemorrhoids based on Goligher’s classification (15); (2) aged between 18 and 80 years. The exclusion criteria were: (1) patients with coagulation disorders, inflammatory bowel disease, incarcerated hemorrhoids, or anal fistula. The study was approved by the ethics committee of the first affiliated hospital of Zhejiang University.

The choice of operative procedure depended on the patient’s own decision. Patients with grade III hemorrhoids who underwent MTL were recruited to the MTL group, and patients who underwent SH group were recruited to SH group and selected by propensity score matching (PSM).

### MTL and SH

All surgeries were performed in the prone jackknife position with caudal or epidural block or spinal anesthesia by the same surgical team. In the MTL group, a triple thread hemorrhoid ligator (ZDFR Medical Science and Technology Ltd., Guangzhou, China) was applied and self-assembled with three elastic threads. The MTL included the following steps: (1) A prolapse test was performed using gauze and an anoscope. (2) Ligation was performed in two levels—the first level was placed on the up pole of the internal hemorrhoids, and the second level was placed on the internal hemorrhoid tissue. The maximum number of ligation points on the same layer was three, and approximately 0.5 cm mucosal bridge was preserved to prevent anorectal stenosis. During the ligation process, a routine digital vaginal examination was recommended to prevent a rectovaginal fistula, and a digital rectal examination was performed to avoid anorectal stenosis. (3) The elastic threads were released to ligate loose rectal mucosa at –0.08 MPa. (4) For some smaller hemorrhoids near the dental line that were not suitable for elastic thread ligation, 0^#^ MERSILK sutures (Ethicon Inc., Somerville, NJ, USA) were employed for ligations, assisted by a hand-made knot pusher.

In the control group, SH was performed using CPH32 (Frankenman International Ltd., Hong Kong, China) according to a standardized methodology (16).

The patients in both groups were treated with antibiotics intravenously for 1 day and orally for 3 days. All patients were given oral laxatives for 1 week. It is recommended that the patient take a sitz bath after defecation.

### Outcomes

The primary outcome was the recurrence of prolapse after 6 months of follow-up. Secondary outcomes were operative time, post-operative pain scores, hospital stay, the incidence of complications, Wexner incontinence score (17) at day 1 postoperative, and quality of life of patients with constipation. Recurrence was based on the findings of the patient’s complaint of a reappearing prolapse and the surgeon’s examination. The Wexner incontinence scoring system cross-tabulates frequencies (Never = 0/Rarely = 1/Sometimes = 2/Usually = 3/Always = 4) and different anal incontinence presentations (Gas/Liquid/Solid/Pad use/Need for lifestyle alterations) and sums the returned score to a total of 0–20 (where 0 = perfect continence and 20 = complete incontinence) (17).

Patient demographics, operative data, and follow-up information were recorded. Follow-up was performed to record pain score of the 1st post-operative day (using a Numeric Rating Scale), Wexner incontinence score 6 months after operation, and postoperative complications (including urinary retention, bleeding, anorectal stenosis, and rectovaginal fistula). Patients’ constipation symptoms and constipation-related quality of life were re-evaluated after 6 months of follow-up. Patients’ constipation symptoms were evaluated using the Patient Assessment of Constipation-Symptoms (PAC-SYM) questionnaire (18), and patients’ constipation-related quality of life was assessed using the Patient Assessment of Constipation-Quality of Life (PAC-QOL) questionnaire (19).

### Statistical analysis

The statistical software package SPSS 23.0 (IBM Corp., Armonk, NY, USA) and GraphPad Prism 6.02 (GraphPad Software, San Diego, CA, USA) was used for statistical analysis. Patients who underwent SH were selected by PSM with a ratio of 1:1, matched based on age, sex, incontinence score, baseline PAC-SYM and baseline PAC-QOL. Kolmogorov-Smirnov and Shapiro-Wilk tests were used for testing normality. Continuous data that confirmed to normal distribution are expressed as mean ± standard deviation (SD) and compared by independent-samples *t*-test. Continuous data that confirmed to skewed distribution are expressed as medians (interquartile ranges) and compared by Mann-Whitney *U* test. Categorical data were expressed as n and compared using Pearson’s χ^2^ test or Fisher’s exact probability test. Two-sided *P* < 0.05 was considered as statistically significant.

## Results

There were 128 patients in the MTL group and 141 patients in the SH group before PSM (Table 1). After matching there were 115 patients eligible for inclusion in the MTL group, and 115 patients who underwent SH were selected by PSM. The two groups of patients had comparable demographics after matching (Table 2).

**TABLE 1:** Pre-operative demographic characteristics of patients before propensity score matching (PSM).

Characteristics	MTL group (*n* = 128)	SH group (*n* = 141)	*P*
Age	42 (36–58)	44 (34–66)	0.226
Gender	–	–	0.034
Male	57	81	–
Female	71	60	–
Incontinence score	0 (0–1)	0 (0–1)	0.347
PAC-SYM	11 (8–12)	10 (7–13)	0.447
PAC-QOL	51 (41–68)	59 (45–71)	0.030

Data are presented as median (interquartile range) values unless otherwise specified. PSM, propensity score matching; MTL, multiple thread ligations; SH, stapled hemorrhoidopexy; PAC-SYM, patient assessment of constipation-symptoms; PAC-QOL, patient assessment of constipation-quality of life.

**TABLE 2:** Pre-operative demographic characteristics of patients after propensity score matching (PSM).

Characteristics	MTL group (*n* = 115)	SH group (*n* = 115)	*P*
Age*	43 (37–57)	45 (35–64)	0.671
Gender*	–	–	0.269
Man	55	61	–
Women	65	54	–
Incontinence score*	0 (0–1)	0 (0–1)	0.768
PAC-SYM*	11 (9–12)	10 (7–12)	0.479
PAC-QOL*	55 (45–67)	56 (46–68)	0.113

*Matched parameters data are presented as median (interquartile range) values unless otherwise specified.

PSM, propensity score matching; MTL, multiple thread ligations; SH, stapled hemorrhoidopexy; PAC-SYM, patient assessment of constipation-symptoms; PAC-QOL, patient assessment of constipation-quality of life.

For the MTL procedure, the median number of ligations was 5 (4–6). Within 6 months of postoperative follow-up, the MTL technique resulted in five cases of recurrence, and the SH technique resulted in seven cases of recurrence (*P* = 0.352). Patients’ constipation symptoms and quality of life between the MTL and SH groups remained comparable, as evaluated by PAC-SYM and PAC-QOL, respectively, at 6 months after surgery (*P* > 0.05). The median operative time was 16 min (15–18 min) in the MTL group versus 25 min (16–33 min) in the SH group (*P* < 0.01). The median length of hospital stay was 2 d (1–3 d) in the MTL group versus 2 d (2–2 d) in the SH group (*P* > 0.05). On the 1st postoperative day, there were no significant differences in pain scores between the two groups (*P* = 0.658) (Table 3).

**TABLE 3:** Post-operative and follow-up details of patients after propensity score matching (PSM).

Characteristics	MTL group (*n* = 115)	SH group (*n* = 115)	*P*
Recurrence (6 months), (*n*)	5	7	0.352
Operative time (minutes)	16 (15–18)	25 (16–33)	<0.001
Pain score (1st PD)	2 (1–3)	2 (2–3)	0.658
Hospital stay (days)	2 (1–3)	2 (2–2)	0.067
**Complications (*n*)**			
Urinary retention	18	21	0.598
Bleeding	1	8	0.035
Anorectal stenosis	1	5	0.213
Rectovaginal fistula	0	0	0.999
Incontinence score (6 months)	0 (0–1)	0 (0–1)	0.571
PAC-SYM (6 months)	7 (4–10)	8 (6–10)	0.093
PAC-QOL (6 months)	50 (41–60)	53 (40–63)	0.228

PD, post-operative day. Data are presented as median (interquartile range) values unless otherwise specified. MTL, multiple thread ligations; SH, stapled hemorrhoidopexy; PAC-SYM, patient assessment of constipation-symptoms; PAC-QOL, patient assessment of constipation-quality of life.

There were five cases of anorectal stenosis in the SH group compared with one in the MTL group (*P* = 0.213), the stenosis was relieved by repeated digital rectal dilatation. There were no cases of rectovaginal fistula in either group (Table 2). Univariate analysis showed that the MTL technique was associated with a lower risk of postoperative bleeding compared with the SH technique (*P* = 0.035).

## Discussion

This study indicated that MTL and SH resulted in comparable outcomes in recurrence rates, postoperative pain, hospital stay, Wexner incontinence scores, and constipation-related quality of life; In terms of complications, there was no significant difference in urinary retention, anorectal stenosis and rectovaginal fistula between the two groups, nevertheless, MTL was associated with less risk of surgical bleeding than SH. These results suggest that MTL had comparable outcomes compared with the technique of SH for the management of grade III hemorrhoids, and MTL seemed to have an advantage of less surgical bleeding.

Hemorrhoids are three columns of vascular tissue, smooth muscle, and connective tissue lining the anal canal (6). In healthy people they provide cushions that maintain continence (2). However, the term hemorrhoid has become synonymous with hemorrhoidal disease. The usual treatment of hemorrhoid is guided by their grade (4). RBL has evolved into an effective therapeutic method for the management of grade I/II hemorrhoids when basic therapy fails (7, 8). For patients with grade III hemorrhoids, hemorrhoidectomy is often the first treatment of choice, and RBL has been regarded as an alternative treatment (7). Currently, the widely accepted theory of the pathophysiology of hemorrhoids is the cushion theory (20). RBL conforms to the cushion theory for the treatment of patients with hemorrhoids; however, the technique of RBL seems imperfect, and its superiority over conventional hemorrhoidectomy or SH has not been proven for the management of grade III hemorrhoids (3). The reasons may lie in the following aspects: firstly, for RBL, only internal hemorrhoids are ligated without fixation of the redundant rectal mucosa. For grade III hemorrhoids, it could be postulated that since both internal hemorrhoids and rectal mucosa have prolapsed, both internal hemorrhoid tissue and redundant mucosa should be fixed; thus, multiple ligations are usually required. Secondly, for RBL, the rubber bands are prone to fall off at an early stage, which might lead to delayed hemorrhage.

The MTL technique may overcome the drawbacks of RBL for grade III hemorrhoids. Firstly, in contrast to RBL, in the current study, we placed two levels of ligations for fixation of not only the loose rectal mucosa but also the swollen internal hemorrhoid tissue, and the median number of ligations was five. Secondly, in addition to elastic thread ligations, 0^#^ MERSILK threads were manually placed for ligation for some smaller internal hemorrhoids when elastic ligations were not suitable as the elastic thread were too thick. This modification indicated a potential economic benefit of this technique. Further, the principle of MTL seems to be similar to that of the Gant-Miwa procedure, which is a therapeutic option for full-thickness rectal prolapse (21).

Safety is of paramount importance in treating hemorrhoids. Compared with SH, by univariate analysis, the incidence of postoperative bleeding of MTL was significantly decreased (*P* < 0.05). During SH, a ring of rectal mucosa above the internal hemorrhoid was extracted, and disastrous complications had been reported due to perforation or bleeding from the anastomosis (13); Mild and moderate anastomotic bleeding during operation was also common, which often required some time for suturing hemostasis, and this could explain why the technique of MTL resulted in less operative time than that of the technique of SH. During RBL, it has been suggested to inject sclerosant agents into the ligated hemorrhoid sac empirically to prevent early fall-off of the rubber bands on the internal hemorrhoid tissue. During MTL, hemorrhoidal tissues could be ligated tightly using threads; thus, the risk of acute or delayed bleeding could be minimized and the operation time could be shortened subsequently, and only one case of delayed hemorrhage was reported due to the fall off of the ligated thread.

There are some limitations to this study. Firstly, for the MTL group, no pathologic specimen was obtained, and the technique cannot be accomplished by only one surgeon as an office-based procedure. Although comparable Wexner incontinence scores were reported, anorectal manometry and transanal endosonography were not routinely performed to evaluate the adverse effects of the two procedures. Secondly, because of the retrospective nature of the study, selection bias seems inevitable. Moreover, the study sample was relatively small, and some data were supplemented by phone follow-up, so measurement bias should also be considered. A multicenter randomized controlled study with enlarged population is necessary in future to fully evaluate any safety benefits with MTL.

In conclusion, the MTL technique might achieve comparable operative outcomes compared with the SH technique for the management of grade III hemorrhoids, nevertheless, MTL seemed to be associated with less risk of surgical bleeding than SH.

## Data availability statement

The raw data supporting the conclusions of this article will be made available by the authors, without undue reservation.

## Ethics statement

The studies involving human participants were reviewed and approved by the First Affiliated Hospital, College of Medicine, Zhejiang University. The patients/participants provided their written informed consent to participate in this study. Written informed consent was obtained from the individual(s) for the publication of any potentially identifiable images or data included in this article.

## Author contributions

XZ wrote the main manuscript text. FL, CL, WC, and JX prepared tables. All authors contributed to the article and approved the submitted version.
